# Multi-vehicle adaptive 3D mapping for targeted ocean sampling

**DOI:** 10.1371/journal.pone.0302514

**Published:** 2024-05-08

**Authors:** Tore Mo-Bjørkelund, Sanna Majaneva, Glaucia Moreira Fragoso, Geir Johnsen, Martin Ludvigsen

**Affiliations:** 1 Department of Marine Technology, Norwegian University of Science and Technology, Trondheim, Norway; 2 Department of Biology, Norwegian University of Science and Technology, Trondheim, Norway; 3 Arctic Biology Department, University Centre in Svalbard (UNIS), Longyearbyen, Norway; 4 Arctic Technology Department, University Centre in Svalbard (UNIS), Longyearbyen, Norway; University of Maine, UNITED STATES

## Abstract

Expanding spatial presentation from two-dimensional profile transects to three-dimensional ocean mapping is key for a better understanding of ocean processes. Phytoplankton distributions can be highly patchy and the accurate identification of these patches with the context, variability, and uncertainty of measurements on relevant scales is difficult to achieve. Traditional sampling methods, such as plankton nets, water samplers and *in-situ* vertical sensors, provide a snapshot and often miss the fine-scale horizontal and temporal variability. Here, we show how two autonomous underwater vehicles measured, adapted to, and reported real-time chlorophyll a measurements, giving insights into the spatiotemporal distribution of phytoplankton biomass and patchiness. To gain the maximum available information within their sensing scope, the vehicles moved in an adaptive fashion, looking for the regions of the highest predicted chlorophyll a concentration, the greatest uncertainty, and the least possibility of collision with other underwater vehicles and ships. The vehicles collaborated by exchanging data with each other and operators via satellite, using a common segmentation of the area to maximize information exchange over the limited bandwidth of the satellite. Importantly, the use of multiple autonomous underwater vehicles reporting real-time data combined with targeted sampling can provide better match with sampling towards understanding of plankton patchiness and ocean processes.

## Introduction

Marine phytoplankton are responsible for approximately half of the global oxygen production [1]. However, accurately measuring and mapping phytoplankton blooms poses a significant challenge using conventional sampling techniques. Previous attempts to map the dynamic conditions of these blooms have utilized autonomous underwater vehicles (AUVs) equipped with adaptive sampling algorithms [2–4]. These algorithms allow the vehicle to adjust its path based on real-time measurements gathered during its operation, adapting their path to gather the most useful information. However, prior efforts have been limited to two-dimensional modeling of the environment and have often assumed a static underlying field. In contrast, our proposal aims to advance these methodologies by expanding modeling capabilities into three dimensions and incorporating temporal effects. This expansion enables a more comprehensive understanding of the complex dynamics of phytoplankton blooms. Moreover, increasing the number of deployed autonomous vehicles facilitates higher-resolution data collection. This elevated granularity empowers operators and vehicles alike with contextual information for informed decision-making in the field.

Adaptive robotic sampling, using chlorophyll a estimated from fluorescence (CF) as a proxy for phytoplankton concentrations in “hot spots” [2] has been suggested as a solution to provide information on their patchiness. CF measured in live phytoplankton cells using *in-situ* sensors can be used as a biomass proxy, but the values must be verified from *in-vitro* analyses of chlorophyll a from water samples. Therefore, the combination of adaptive robotic sampling and physical sampling of phytoplankton is needed [3, 5]. However, by not sharing the data collected by the AUVs in real time, the ocean sampling is still done deterministically. Adaptive sampling, using AUVs for targeted ocean measurements [2, 4, 6–14], is a commonly used method for capturing dynamic and transient phenomena. In adaptive methods, path planning is often done by *deliberation* by a utility function evaluated along potential paths. The utility function needs a prediction of the distribution of the environmental variables of interest, such as salinity, temperature, and CF. Common modeling approaches are Gaussian processes (GP) [15] and higher-order simulation models [16]. Estimation of the horizontal distribution of Chlorophyll a using a *log*-GP (*ℓ*GP) has been explored previously [2, 4]. They also explored multi-vehicle communication using asynchronous surfacing.

While phytoplankton distributions are patchy and unpredictable, they are not without structure or spatial correlation. *ℓ*GP modelling can exploit the spatial correlation in phytoplankton patches and attempt to estimate the CF at unmeasured locations while accounting for the patchy “hot-spot” nature of algal blooms [2].

The exploration problem with multiple sampling platforms was formalized in a sequential decision-theoretic planning under the uncertainty framework termed the multirobot adaptive sampling problem [2]. For efficient multi-vehicle adaptive sampling, data sharing between agents is essential for mission cooridnation and on-board model error reduction [17]. Communications-constrained multi-vehicle sampling was investigated and showed that error reduction of the on-board model is dependent on the quality of the communication between agents [17]. Communication-constrained multi-vehicle operations were carried out off the California coast, using a fleet of AUVs, communicating over the horizon by IRIDIUM^®^ [18, 19], the Autonomous Ocean Sampling Network-II deployed a multi-month mission to map coastal waters [20], in an effort to develop a sustainable, portable, and adaptive ocean sampling system. The Adaptive Sampling And Prediction (ASAP) project [21] set out to monitor and coordinate a set of gliders using over-the-horizon communication.

Phytoplankton, as the trophic base of almost all marine ecosystems, acts as a climate regulator by converting CO_2_ to organic matter through photosynthesis, which can ultimately be exported to the deep ocean [22, 23]. Their community composition and timing of events can be sensitive to climate change and environmental perturbation, impacting higher trophic levels, which can eventually cause an irreversible shift in the ecosystem [24, 25]. The spatial distributions of phytoplankton are highly heterogeneous, or patchy, [26] and high density patches can occur at a wide range of spatial scales, from mesoscales to microscales [27]. The patchiness of phytoplankton is driven by physical, chemical, and biological processes [27–30]. The horizontal extent of these patches can be observed in surface waters using satellite images [31, 32]. Phytoplankton patchiness can exhibit as subsurface chlorophyll maxima, which is measured by vertical profiling sensors. Taking spurious vertical profiles limits the three-dimensional understanding of patchiness below the surface. Traditional ship-board net or water sampling for phytoplankton at predetermined locations and depths provides integrated, or averaged, data that can miss the high resolution structure of phytoplankton community structure, composition, and abundance. In the Arctic, the common haptophyte *Phaeocystis pouchetii* can occur as single cells or colonies up to three centimeters in diameter [33], as well as forming high-density blooms [24]. These colonies and blooms are often not fully resolved in space and time due to a lack of sufficient resolution in the sampling.

The goal of this work is to provide a method for informed physical sampling of Chlorophyll a and contextualize the samples. By adapting the vehicle path towards high concentrations of CF, the depth of the highest values of CF within an area can be estimated and sampled. Adaptive informative sampling can have an advantage over traditional “lawnmower” surveys [34]. An adaptive “uncertainty-aware” method was suggested [34], where a simulated vehicle attempted to synoptically map the distribution of phytoplankton off the coast of California. Their main finding was that the adaptive algorithm had an advantage over the classic “lawnmower” survey in reducing the uncertainty of the estimation, reducing biases incurred from human-designed surveys.

As spatiotemporal patchiness plays a fundamental role in the functioning of marine ecosystems [27], understanding the nature of this process is needed to disentangle predator-prey interactions and the flow of carbon through the food web. Here, we show how two AUVs reporting real-time data for targeted discrete water sampling can provide a better match with sampling actions and fine-scale plankton patchiness. Additionally, by having the AUVs adapt their path and share measurements, they are able to effectively map the three-dimensional extent and temporal variability of the patchy phytoplankton bloom, as tested in simulation (Fig 4). By combining adaptive robotic sampling with over the horizon satellite communication and a manned vessel, we created a heterogeneous sampling network for targeted sampling during a spring phytoplankton bloom (May 2022) in Kongsfjorden, Svalbard (Fig 5a and 5b). Adaptive sampling is achieved by modeling CF on board the AUVs using a *ℓ*GP [2], in three dimensions, with the AUVs seeking regions of high CF and high uncertainty, following the sense-plan-act cycle [35]. Data is exchanged through the network over the IRIDIUM^®^ [18, 19] satellite link and displayed to the operators in a central operator hub. The human-crewed vessel acted on the information gained by the autonomous agents to take targeted and contextualized water samples. This three-dimensional reconstruction of phytoplankton CF patchiness evolving in space and time offers a unique opportunity to understand the mechanism underlying their heterogeneous distributions.

## Materials and methods

**Fig 1:**
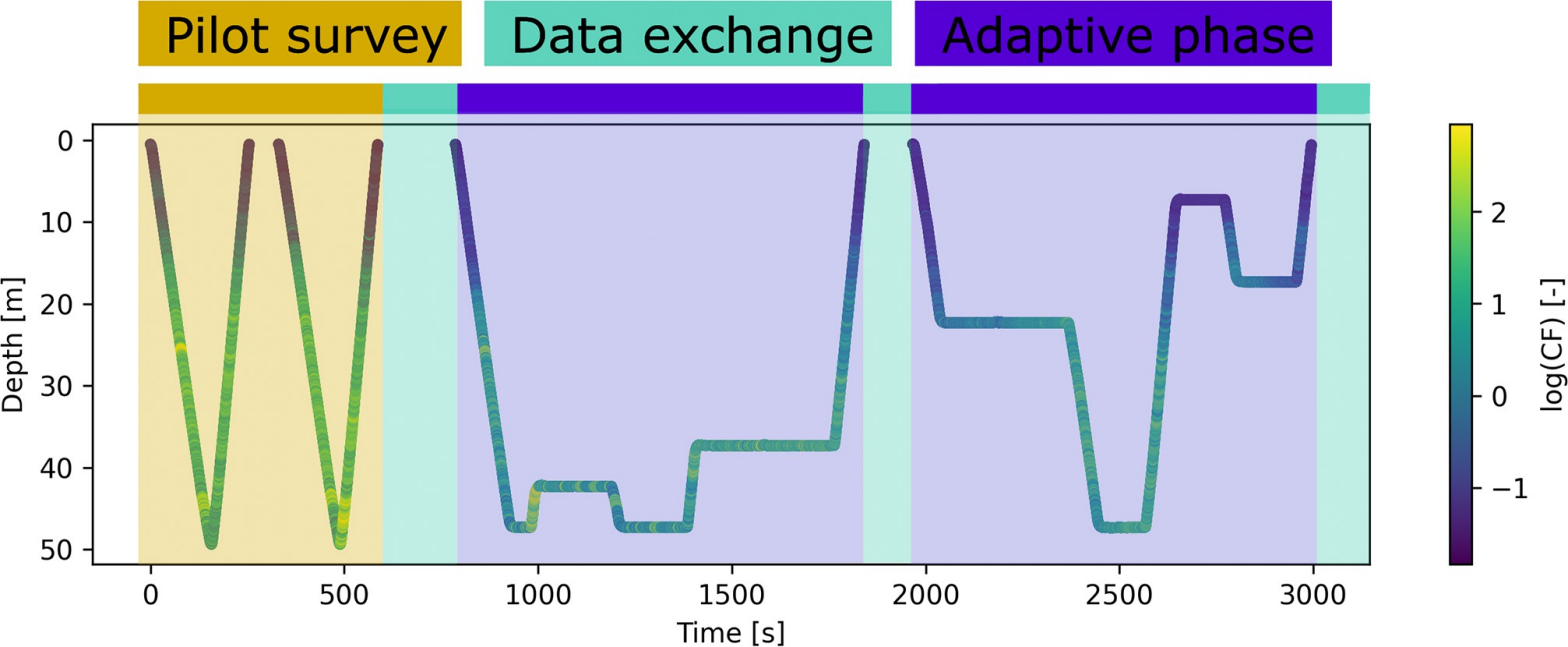
Timeline of LAUV Roald on the 28th of May 2022, depth plotted against time, with color indicating the value of CF, plotted as log(CF) for visualization.

The method was built on the *onboard algorithm* used for adaptive behavior and data exchange, developed using simulation and field tested. The algorithm *on board* consists of (a) the predefined *pilot survey*, (b) the *data exchange*, and (c) the *adaptation phase*. The *adaptive phase* was further subdivided into (i) the on-board modelling and (ii) the path planning. For the AUVs, the mission was initialized by executing a pilot survey, followed by a data exchange. After this initial phase, the adaptive phase ran for a fixed period before a data exchange action was performed and the adaptive phase started again (Fig 1). The *sampling network* used the data collected by the AUVs for informed sampling from a manned craft. Adaptive path planning was carried out onboard the autonomous vehicles, providing robustness to communication loss and enabling *in-situ* decision making. No particular permits were needed for the experiments, as they were carried out in Norwegian territorial waters.

### On-board algorithm: Pilot survey

A rectangular cuboid was selected as the operational *volume* shape for exploration, and the operational *area* is the projection on the horizontal plane of this volume. The size and orientation of the volume is commonly decided by the operator, depending on the nature of the mission, the number of vehicles available, their speed and the desired accuracy of the spatiotemporal model [20]. The *pilot survey* [4], is usually a pre-planned path, designed to gather data for the initialization of the on-board model. In this case, we set it as a crossing of the operational area by all AUVs in the system, where the path is determined by the number of vehicles, *N*_*v*_, in the network. All vehicles were set to cross from *y* = 0 to *y*_*max*_ in parallel paths. The *x* value of the path was determined by

$$\begin{array}{c} x_{v}=V_{x}\frac{2\cdot n_{v}+1}{2\cdot N_{v}} \end{array}$$ (1)

for vehicle *n*_*v*_ of *N*_*v*_, where *n*_*v*_ is 0-indexed, and *V*_*x*_ was the x-extent of the operational area. The *pilot survey* path undulated between the surface and the maximal depth of the operational volume. After the *pilot survey*, the data collected was segmented and sent to the *operator hub* in a surfacing event, where the vehicle received data available from the other vehicles in a *data exchange* phase.

### On-board algorithm: Data exchange

As data is collected at high rates (>1Hz) on different platforms moving at nominal speeds of 1.0–1.5m/s, it was segmented to fit the predefined message structure; enabling computational feasibility of the on-board model and data sharing over low bandwidths. The segmentation algorithm used the shared knowledge of the agents in the operational area, whereas the *data exchange* used segmented data for efficient low-bandwidth communication.

#### Segmentation

For computational feasibility of the on-board model, the number of samples usually needs to be restrained to below ∼10000 [15]. Measurements were segmented into a *n*_*x*_ × *n*_*y*_ × *n*_*z*_ grid, reducing the maximum sample size to *n*_*x*_ ⋅ *n*_*y*_ ⋅ *n*_*z*_ and creating a 3D grid in the operational volume. The number of grid cells was decided by the operator and could be adjusted according to desired spatial resolution and the available on-board computing power. All agents shared a commonly defined grid. The measurements collected were assigned to their respective grid cell. The data value of that cell was set to the average of the measurements taken within that cell since the last segmentation. Previous measurements were discarded if new data was collected within that cell. We assumed that temporal de-correlation would make previous measurements irrelevant compared to the new measurements. Cells with previous held data values and no new measurements kept their data intact. Data segmentation was performed at each update step, before evaluation of the on-board model and adaption.

#### Exchange

**Fig 2:**
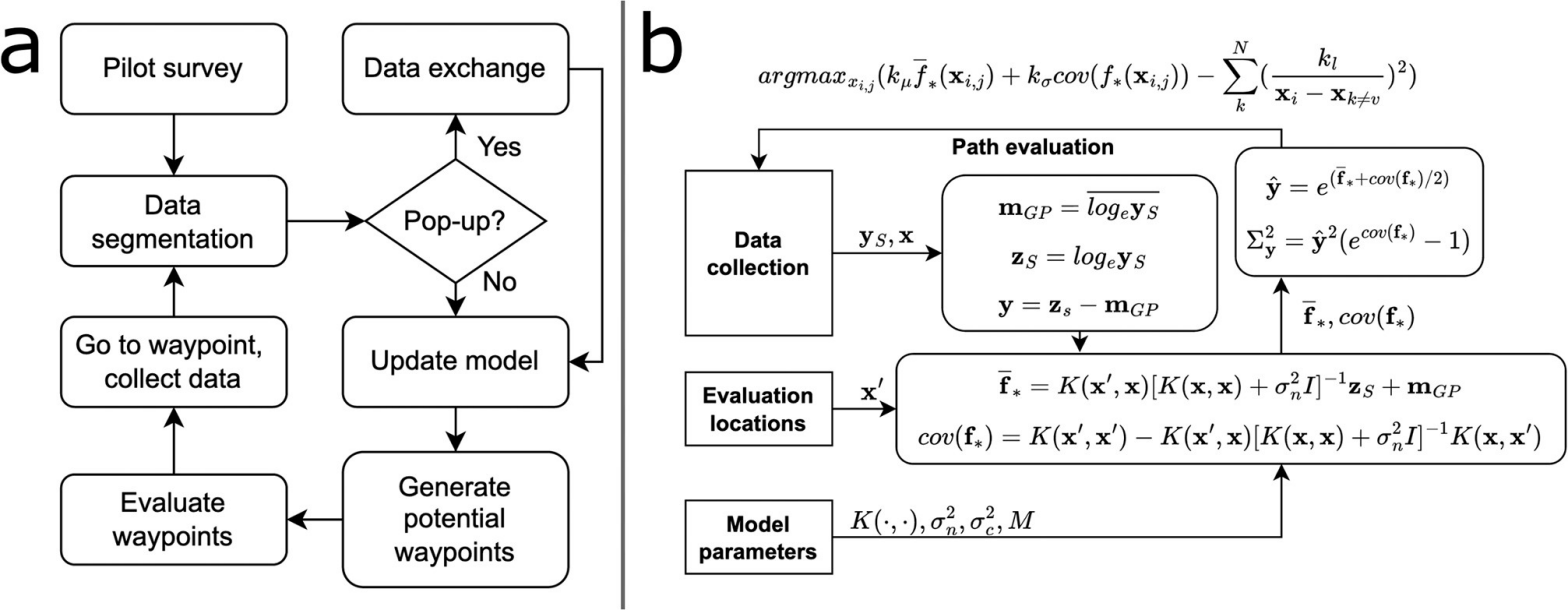
Overview of the *on-board algorithm*. Flowcharts of the (a) *on-board algorithm*, starting at the *pilot survey*, followed by the cyclical *data exchange* and *adaptive phases*, and (b) *adaptive phase*, with data ingestion, *ℓ*GP evaluation at potential waypoints, **x**′, and their evaluation.

The AUVs surfaced at regular intervals, transmitting the latest segmented data, following the flowchart (Fig 2a). The surfacing event served two main purposes, (1) to exchange data with the other vehicles in the system and (2) to obtain a GPS fix to bound the navigational uncertainty of the vehicle. Data, vehicle position, and time were transmitted by IRIDIUM^®^ [18, 19] short burst data (SBD) to a central *operator hub*, where the operator monitored the operation and data flow. The *operator hub* then responded to the vehicle with the most recent data and positions received from the other agents in the system. The IRIDIUM^®^ SBD protocol limits the message size to 340 bytes [18, 19], while the vehicle-specific driver needed 20 bytes for overhead. Data for one grid cell took up *n*_*d*_ = 3 + *n*_*c*_ bytes, where *n*_*c*_ is the number of data channels (e.g. fluorescence, dissolved oxygen, temperature, salinity), creating the message structure (Table 1). This enabled the sending of up to $l_{d}=\left\lfloor \frac{320}{n_{d}}\right\rfloor$ data points over IRIDIUM^®^ SBD.

**Table 1:** Overall message structure with the header in the first 10 bytes and the data in the remaining, depending on the available data and SBD limitations. Data message structure for *n*_*c*_ = 1, cell number is sent as a UInt16, as the number of grid cells should be in the interval 256 to 65536, and where *k* ∈ [0, …, *l*_*d*_) is the data index.

Field	message ID	vehicle ID	lat	lon	data	Cell no.	Time
Start byte	0	1	2	6	10 + *kn*_*d*_	11 + *kn*_*d*_	13 + *kn*_*d*_
Data type	UInt8	UInt8	Float32	Float32	UInt8	UInt16	UInt8

If the queue of unsent data points was longer than *l*_*d*_, the message was truncated to contain the *l*_*d*_ newest data points. The *operator hub* also sent a message containing the last known positions of the other vehicles in the network to the surfaced vehicle. This message was not as constrained in size as the data message, since the number of vehicles, *N*_*v*_, is sufficiently small.

#### Operator hub

As the communication center in the sampling network, the *operator hub* distributed the information throughout the sampling network, both to the AUVs and operators. Data was transmitted to the *operator hub* and presented to the operators both as raw messages, and the *ℓ*GP realization, with the latest vehicle position. From there, the operators could interrogate the model for a predicted profile anywhere in the operational space along with its error bounds. Based on this, operators could choose to act on the information and perform targeted sampling, having a greater understanding of the contextual data field. The act of sending the data near real time through the operator hub enabled a ‘human-in-the-loop’ approach. When the data exchange was completed, the vehicles updated the model and began the adaptive phase.

### On-board algorithm: Adaptive phase

In the adaptive phase (Fig 2b), the available data was used as input for a GP [15], in the form of a *ℓ*GP [2, 4]. Potential paths were generated from a set of potential waypoints around the vehicle and deliberated upon using the predictive CF mean value and variance at the waypoint. Furthermore, its proximity to other vehicles in the area was taken into account. When the vehicle reached the desired waypoint, a new waypoint was chosen by the same means until it was time for a *data exchange*.

#### On-board model

The *ℓ*GP [2, 4, 15] was expanded to three physical dimensions and time-varying uncertainty was added to the measurements. We denoted the set of measurements **y**_*S*_ at locations *X* at times *τ*, with *S* = [*X*, *τ*] and let **z**_*S*_ = *log*_*e*_**y**_*S*_ − **m**_*GP*_, where $m_{GP}=\bar{log_{e}y_{S}}$ was the mean of the input data. When taking the logarithm of the measurements, they became unitless [36]. We assumed that $z_{S}∼N\left(\mu ,\sigma_{c}^{2}\right)$, such that a GP could be used for predictions of **z** at unobserved locations and times by the formulation in Eqs (2) to (4) [15].

$$\begin{array}{cc} & f_{*}|S,z_{S},S_{*}∼N\left({\bar{f}}_{*},cov\left(f_{*}\right)\right)\text{,}\;\text{where} \end{array}$$ (2)

$$\begin{array}{cc} & {\bar{f}}_{*}≜E\left[f_{*}|S,z_{S},S_{*}\right]=K_{S_{*},S}{\left[K_{S,S}+\sigma_{n}^{2}I\right]}^{-1}z_{s}+m_{GP} \end{array}$$ (3)

$$\begin{array}{cc} & cov\left(f_{*}\right)=K_{S_{*},S_{*}}-K_{S_{*},S}{\left[K_{S,S}+\sigma_{n}^{2}I\right]}^{-1}K_{S,S_{*}} \end{array}$$ (4)

Where S_*_ = [X_*_, *τ*_*now*_] was a set of prediction locations at the time of the prediction, *τ*_*now*_, ${\bar{f}}_{*}$ was the predictive mean at the locations S_*_, and *K*_*u*,*v*_ was the kernel function evaluated at the locations *u* and *v*, and $\sigma_{n}^{2}$ was the nugget variance [37]. The kernel function (Eq 5), where *M* = *diag*([*M*_*x*_, *M*_*y*_, *M*_*z*_, *M*_*τ*_]) is the diagonal matrix of de-correlation lengths for each dimension of **s**, including time *τ*, where **s** is a row of *S*; one location.

$$\begin{array}{c} k\left(s,s^{\prime }\right)=\sigma_{c}^{2}e^{-\frac{1}{2}{\left|\frac{s-s^{\prime }}{M}\right|}^{2}} \end{array}$$ (5)

Time was included in the kernel function to account for the temporal decay of accuracy in the measurements. This lead to the covariance matrix having to be updated at each evaluation step, further leading to a full evaluation of the GP at each iteration, in stead of step-wise ingestion of data [3, 14]. In order to produce a predictive mean for the value of CF, the predictive mean for **z**_*S*_ must be transformed [2] as presented in Eqs (6) and (7).

$$\begin{array}{c} \hat{y}=e^{\left(f_{*}+cov\left(f_{*}\right)/2\right)} \end{array}$$ (6)

$$\begin{array}{c} \Sigma_{y}^{2}={\hat{y}}^{2}\left(e^{cov(f_{*})}-1\right) \end{array}$$ (7)

From Eqs (6) and (7) we see that the variance, $\Sigma_{y}^{2}$, is dependent on the predictive mean, unlike for the linear GP. This needs to be taken into account when evaluating the potential paths for optimal, adaptive sampling.

#### Path planning

**Fig 3:**
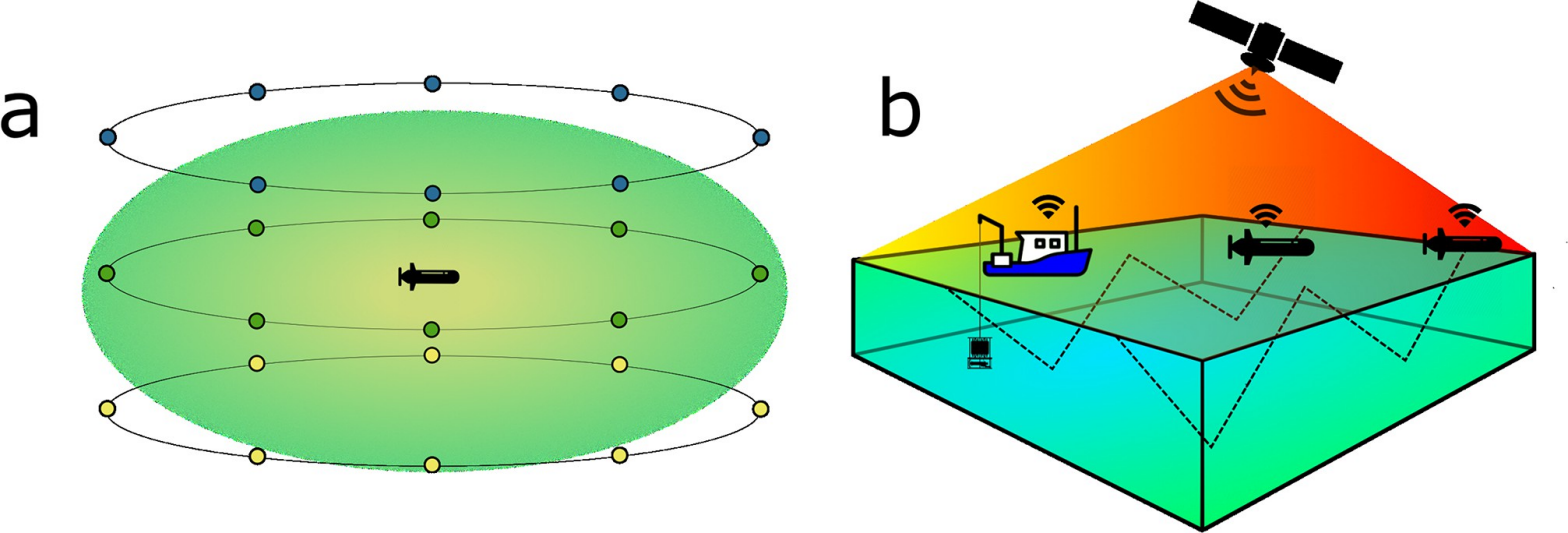
Conceptual illustrations of (a) the potential waypoints generated at every update step by having *n*_*θ*_ = 8 segments, 3 waypoints in the vertical per segment, resulting in 24 potential waypoints around the AUV, and (b) mission setup for the field trials, using two AUVs and a work boat, communicating over satellite.

After data was ingested into the *ℓ*GP, it was evaluated at potential waypoint locations, in order to find the optimal path. Using the current position of the AUV, **x**_*v*_, as the center, it created 3*n*_*θ*_ potential waypoint locations, where *n*_*θ*_ is the number of segments uniformly spread around a circle. These locations were generated as **x**_*i*,*j*_ = **x**_*v*_ + [*r* cos *θ*_*i*_, *r* sin *θ*_*i*_, Δ*z*_*j*_], where Δ*z* = [−*z*_*d*_, 0, *z*_*d*_], **x**_*v*_ is the vehicle position at the current time, *r* is a radius, and *z*_*d*_ is the vertical extent of one grid cell (Fig 3a). Each of the potential locations were screened to see if they were inside the operational volume, and the ones who fell outside were discarded. Then, the predictive mean and uncertainty at the potential locations were evaluated along with the distance from the location, **x**_*i*,*j*_, to the last known location of the other vehicles. The optimal waypoint maximized (Eq 8).

$$\begin{array}{c} argmax_{x_{i,j}}\left(k_{\mu }\hat{y}\left(x_{i,j}\right)+k_{\sigma }\Sigma_{y}^{2}\left(x_{i,j}\right)-\sum_{k}^{N}{\left(\frac{k_{l}}{x_{i}-x_{k\neq v}}\right)}^{2}\right) \end{array}$$ (8)

The constants *k*_*μ*_, *k*_*σ*_, and *k*_*l*_ were configuration parameters, enabling the operator to choose the weight of each element of the evaluation function. Tuning of *k*_*μ*_ and *k*_*σ*_ was done in simulation, where the trade-off between uncertainty reduction and maxima-seeking could be set by the operators. Setting *k*_*l*_ is a function of how close the operator wants the vehicles to come to each other, and in deciding its value, the speed of the vehicles and the size of the operational area needs to be taken into account [20]. Notice that only the horizontal distance between the vehicles was taken into account, as the vertical extent of the operational volume usually is far less than the horizontal. After the optimal waypoint was chosen, the AUV travelled towards it, collecting underway measurements. Then, when the waypoint was reached, the measurements were segmented, the *ℓ*GP evaluated, and a new waypoint chosen.

### Simulation and field deployment

The method was implemented in the Robot Operating System (ROS) [38] and tested using a simulated CF field along with the vehicle simulator in DUNE (DUNE: Unified Navigation Environment) [39]. Constellations of multiple AUVs were simulated using a virtual machine for each vehicle, using the host computer as the operator hub, and emulating satellite communication by reading and writing to a shared directory on the host computer. A simplified simulation, showing the basic functionality is available [40], along with the ROS packages [41]. A field deployment was carried out during the Arctic spring bloom, on the 28th of May of 2022 in Kongsfjorden, Svalbard, focusing on an area around sampling station KB3 [42]. Operator input values (Table 2) for the above presented method were chosen based on simulation trials and prior assumptions [17, 43]. Values are common to simulation and field deployment, except the operational volume *x*- and *y*-extent. De-correlation lengths are softly constrained by the grid cell size and the size of the operational area. Small correlation lengths resulting in a high variance in-homogeneous prediction, whereas high correlation lengths would smooth the predictions, possibly erasing important features. The choice of de-correlation lengths here is based on heuristics. A conceptual overview of the operational setup is presented in Fig 3b.

**Table 2:** Operator inputs, with values used for field trials and their explanation.

Variable	Value	Explanation
[*V*_*x*_, *V*_*y*_, *V*_*z*_]	[1500, 1500, 50]m	Operational volume extent in [x,y,z]-directions.
[*V*_*lat*_, *V*_*lon*_, *V*_*θ*_]	[78N 57.29, 11E 56.85, −45°]	[Latitude, longitude, orientation] of operational volume origin.
[*n*_*x*_, *n*_*y*_, *n*_*z*_]	[15, 15, 10]	Operational volume number of grid points in [x,y,z]-directions.
[*M*_*x*_, *M*_*y*_, *M*_*z*_]	[600, 600, 3]m	De-correlation length in [x,y,z]-directions.
*M*_*τ*_	10000s	De-correlation time.
[*σ*_*c*_, *σ*_*n*_]	[1.6, 0.7]	[Variance,nugget] uncertainties of **z**_*S*_, from previous data [3].
[*k*_*μ*_, *k*_*σ*_, *k*_*l*_]	[1, 1, 300*m*]	Configuration parameters of path evaluation function.

#### Vehicles and sensors

The *on-board algorithm* was implemented on two Light AUVs (LAUVs) [39], using their backseat driver [44]. The LAUV concept was developed at the Underwater Systems and Technology Laboratory at the University of Porto, and commercially produced by OceanScan Marine Systems and Technology Lda. The vehicles, LAUVs “Harald” and “Roald” (AUR-Lab, NTNU), and are equipped with a scientific payload of a CTD sensor, and a chlorophyll a fluorometer. Specifically, LAUV “Roald” is equipped with a RBR Turner Cyclops Fluorometer and an AML Smart X CTD, while LAUV “Harald” carries a WetLabs EcoPuck Triplet, measuring chlorophyll a fluorescence, colored dissolved organic matter and optical backscatter, and a SBE 49 FastCAT CTD. The on-board software, DUNE, (also used for simulation) controls the navigation, sensors, and low-level control and guidance on the vehicle. A manned work boat, R/V Teisten (Kings Bay AS), was used as the platform for targeted sampling. It was equipped with a SAIV SD204 CTD with a Seapoint chlorophyll a *in situ* Fluorometer, 300m-capable winch and 10L Niskin bottle water samplers [46].

#### *In vitro* sampling

A subsurface CF maximum could be biased by *non-photochemical quenching* (NPQ) [45], exhibiting as a subsurface peak, by quenching the signal close to the surface rather than representing the true distribution of chlorophyll a in the water column. We compared CF predictions from *in situ* AUV data and shipborne CF measurements with the *in vitro* concentrations of chlorophyll a from water samples. To validate the data retrieved from sensors, discrete water samples for measurement of *in vitro* chlorophyll a were collected using 10 L Niskin bottles [46] on board the work boat. Sample depths were selected based on layers of relatively high, medium and low chlorophyll a concentrations estimated by the model generated in the operator hub from data from the AUVs. Seawater was filtered (0.5 L) onto Whatman GF/F glass fiber filters [5]. In the laboratory, chlorophyll a was extracted in 100% methanol after 20 hours at −20°C in darkness. The chlorophyll a concentrations were determined using a Turner Designs Trilogy fluorometer (model: 7200–000) following the non-acidification method [47].

## Results

Our approach is evaluated in three parts, (1) by simulating up to four AUVs in a common CF field, (2) by a field experiment, deploying the heterogeneous network and comparing the predictions from the AUV data to measurements and samples taken from a crewed vessel, and (3) by challenging the underlying assumptions of the distribution and statistical properties of the CF field. Hence, the results of convergence time in simulation, model performance compared to field measurements, and the statistics of the CF field are presented.

### Simulation

**Fig 4:**
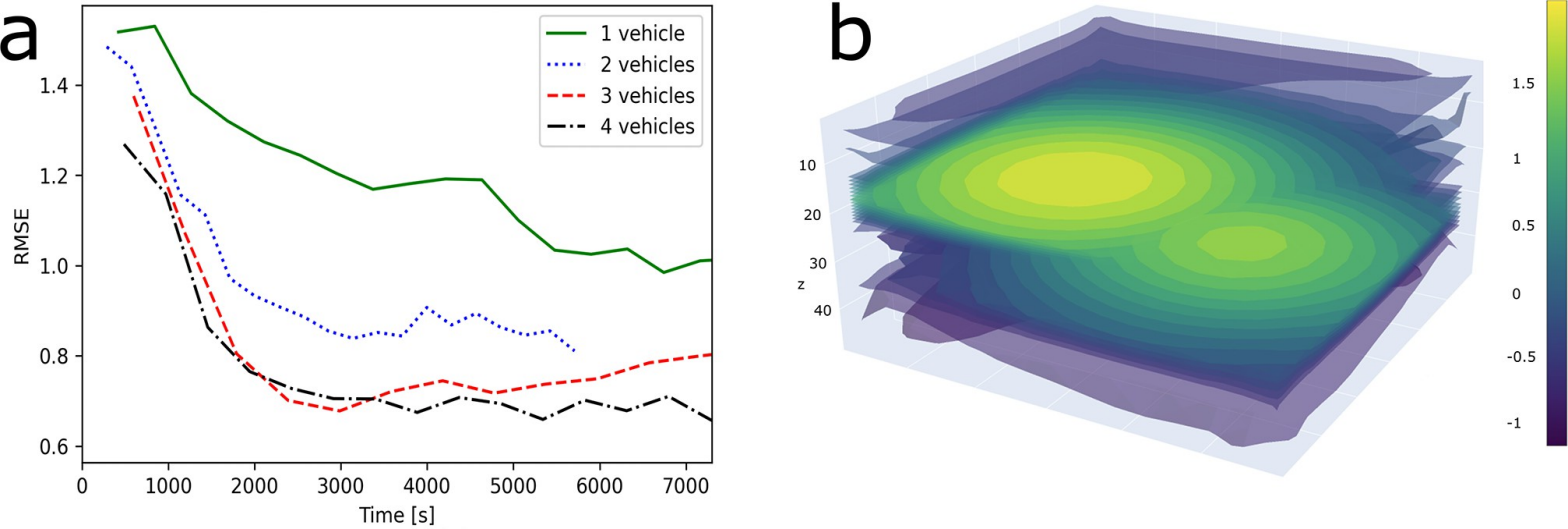
Simulation results and simulated CF field, (a) RMSE of on board models compared to simulated ground truth, of one to four vehicle missions of the same operational area and underlying simulated field. Simulated (b) chlorophyll-a fluorescence field, plotted on a 3000m × 3000m × 50m operational volume used for simulation. Plotted using log-scale for easier visualization.

For development and testing of the algorithm for multi vehicle adaptive mission planning, simulation trials were used, simulating a spring bloom, based on expected values and distributions. The system enabled each vehicle in the heterogeneous network to make decisions *in-situ*, mapping the phytoplankton plume finding a path that minimized uncertainty for the operation volume while seeking CF maxima. A single vehicle simulation over a 3000m × 3000m × 50m volume resulted in the on-board model predictive mean converging after 6000 − 7000 seconds, with the root mean squared error (RMSE) between the on-board model and the simulated field as the convergence criterion. When the single vehicle approach was compared with multi vehicle one, a significant gain in model accuracy and convergence time was observed when adding one vehicle. Further, the benefit of adding more vehicles diminished as the number of vehicles in the network increased. (RMSE results for one to four vehicles, Fig 4a). Using four vehicles, the RMSE converged to the nugget standard deviation of the models used in simulation, *σ*_*n*_ = 0.7.

### Field deployment

**Fig 5:**
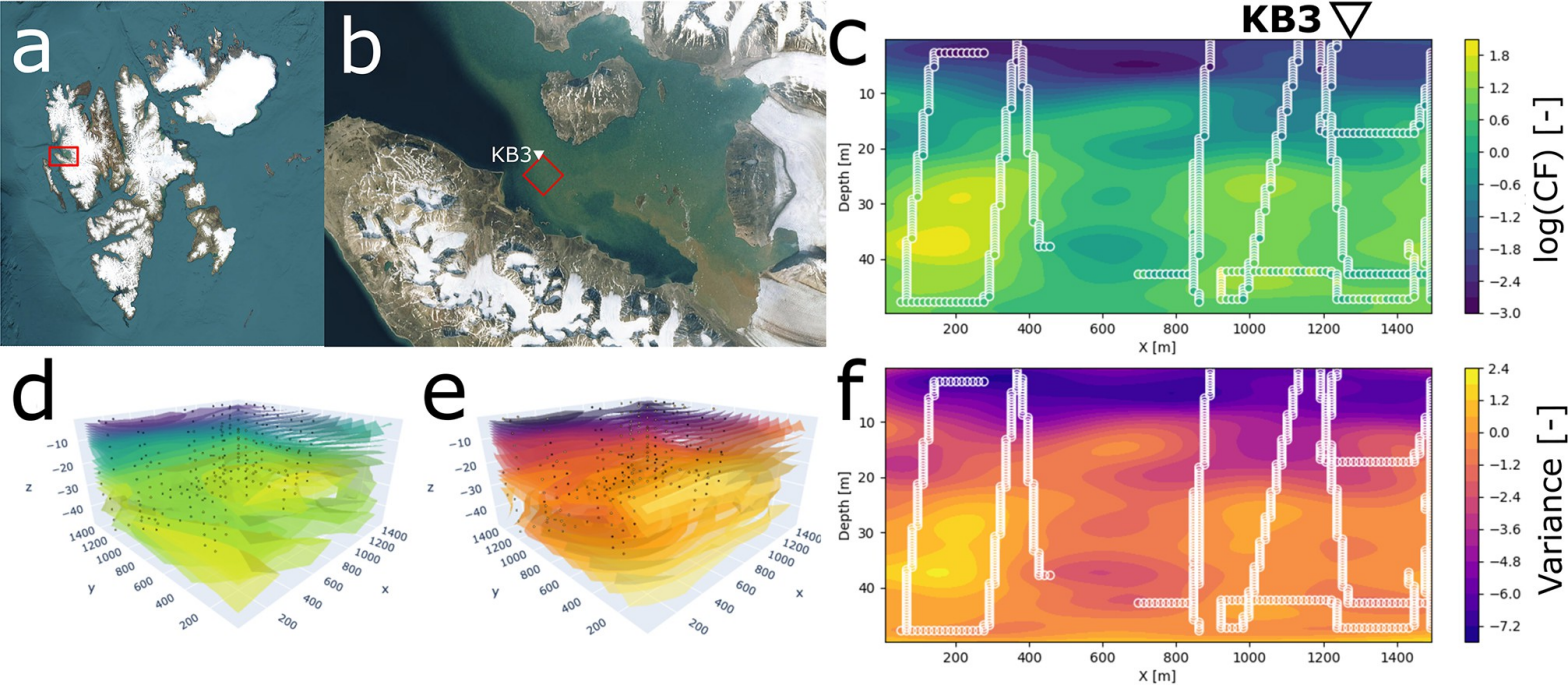
Overview of the operational area of the field trials, and instances of the predicted CF distribution in the operational volume. Satellite overview images of (a) Svalbard (courtesy of U.S. Geological Survey) and (b) Kongsfjorden (courtesy of U.S. Geological Survey), with the operational area marked in red, and station KB3 indicated by a white triangle. Predictive mean CF (c,d), and variance (e,f), of the *ℓ*GP, of the operational volume (c,e) and a horizontal slice (d,f), 300m wide, over the interval *y* ∈ [1200, 1500]m. Plotted in log-scale for visualisation, with circles (c,f) and dots (e,d) indicating grid cells that contain measurements. All field deployment data from AUVs is from 28th of May 2022.

The goal of the field experiment was to estimate the three dimensional distribution of CF in the operational volume by using two AUVs, and to verify the adaptive on-board mapping algorithms and sensing network. Using the CF data from the AUVs, the *ℓ*GP was evaluated at each grid cell in the operational volume (Fig 5d and 5e). A horizontal slice plotted for the interval *y* ∈ [1200, 1500]m (Fig 5c and 5f) to evaluate the area along the Northwestern edge of the operational volume near the point marked KB3. In both cases (Fig 5c–5f), a clear vertical structure in the predicted CF was visible, with a low concentration in the upper 15 − 20m, followed by a layer of higher concentration below 20m. In the horizontal plane, patchiness was also visible with a heterogeneous distribution without a clear trend. There is a sporadic discrepancy between the CF measurements and the underlying field, (a discrepancy feature can be seen in Fig 5c at 45m depth and *x* ≃ 900m), indicating noisy measurements and/or patchy distributions of CF at that position.

**Fig 6:**
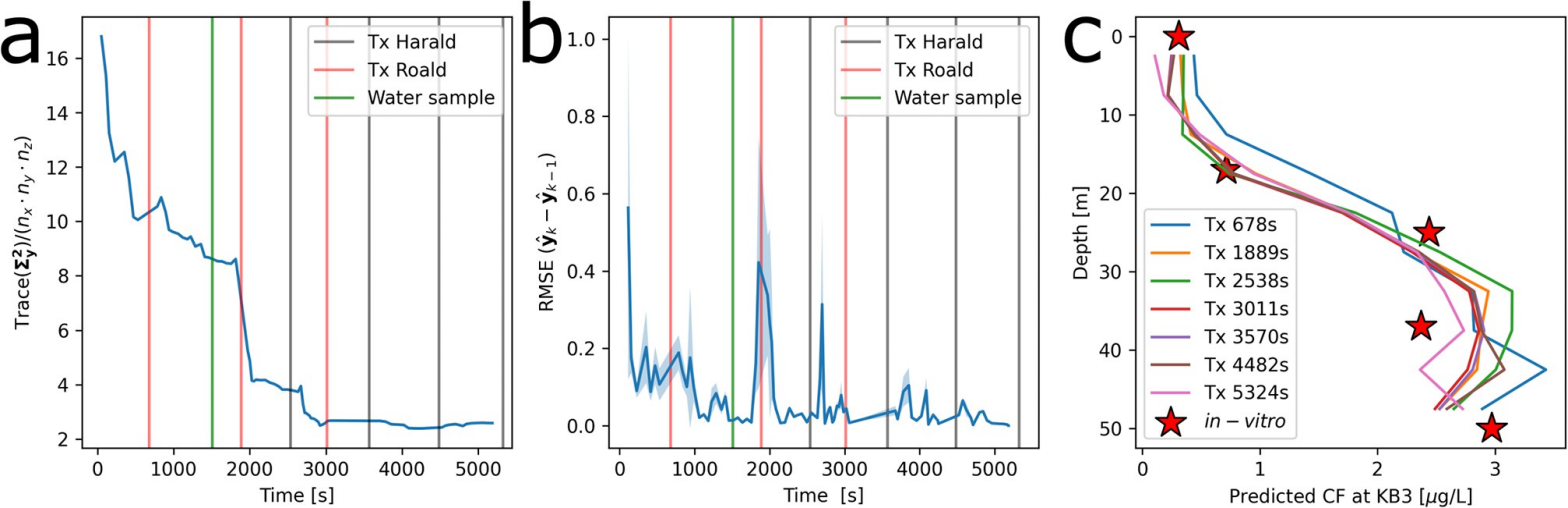
Model performance during field deployment, with water sampling and data exchange sending (Tx) events marked. Averaged (a) prediction uncertainty of each grid cell, (b) prediction error in relation to next prediction for all cells, and synthetic (c) CF profiles at station KB3, after each data exchange event, along with the *in-vitro* chlorophyll a values from water samples. Names (a and b) “Harald” and “Roald” refer to the two AUVs used in the field deployment.

To asses the on-board model, it is re-run in post process, using input from both the AUVs. The averaged model uncertainty for the on-board model decreases for the first 3000 seconds, before leveling off at around 2.5*μg*^2^/*L*^2^ (Fig 6a). In addition to higher precision, the accuracy also increases with time (Fig 6b), with fewer and lower peaks of RMSE between instances of the *ℓ*GP. The predicted CF profiles at station KB3 are plotted after each data exchange event (Fig 6c) as seen from the operator hub. The synthetic profiles follow the same pattern of low CF values in the upper 20m, with a wide maxima between 30 and 50m, showing small variations for the prediction as new data is gathered.

**Fig 7:**
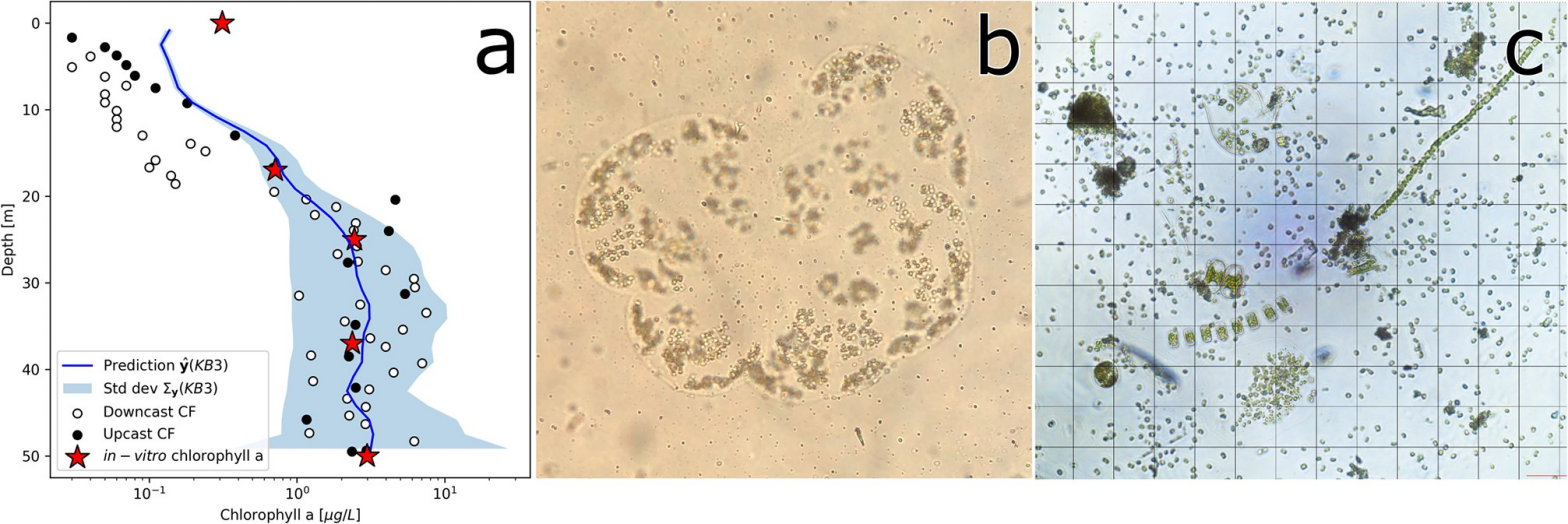
Phytoplankton bloom conditions, (a) a comparison of predicted profile of CF from the *ℓ*GP and its one standard deviation, ship borne CF measurements on both down- and upcast and *in-vitro* chlorophyll a values from water samples at station KB3 at 11:47 local time on the 28th of May 2022. Colony (b) of *Phaeocystis pouchetii* and an (c) example of the phytoplankton community present in the bloom, both (b and c) microscope images. Photos: (b) Ane Cecilie Kvernvik and (c) Anette Wold.

The *ℓ*GP was evaluated at the sampling location (KB3 [42, 48]), producing a predicted CF profile. To validate this profile, it was compared to the *in-situ* CF measurements from the fluorometer attached to the shipborne CTD and the *in-vitro* chlorophyll a values (Fig 7a). These three independent profiles (CF prediction, CF measurement and *in-viro* measurement from water samples) all showed the same trend for the water column. A CTD cast down to 300m showed low CF values near the surface down to 20m, and a subsurface maxima between 28 and 50m. Additionally, the shipborne measurements were noisy, likely caused by the combination of a small sampling volume for the fluorometer and the bloom being dominated by the colony forming *Phaeocystis pouchetii* (colony in Fig 7b and individual cells in Fig 7c). Targeted water samples were taken at locations with both high and low CF predictions based on data from the AUVs (lowest 0 and 17m and highest 25, 37, and 50m). Values of *in-vitro* chlorophyll a from the water samples were approximate to the prediction from the *ℓ*GP (Figs 6c and 7a). In the log-space (Fig 7a), the largest offset between the three profiles is in the upper 15m, where the values are the lowest. The prediction of CF, shipborne CF measurements and *in-vitro* chlorophyll a measurements follow each other closer below 15m. The uncertainty bounds on the predicted CF profile were wide in relation to the noise level on the ship borne CF measurements. This can be caused by the temporal and spatial offset between the AUV measurements and the shipborne measurements combined with the nugget effect noise. This indicates that the *ℓ*GP was well configured and able to predict the *in-situ* and *in-vitro* values and uncertainties.

### Statistical properties

**Fig 8:**
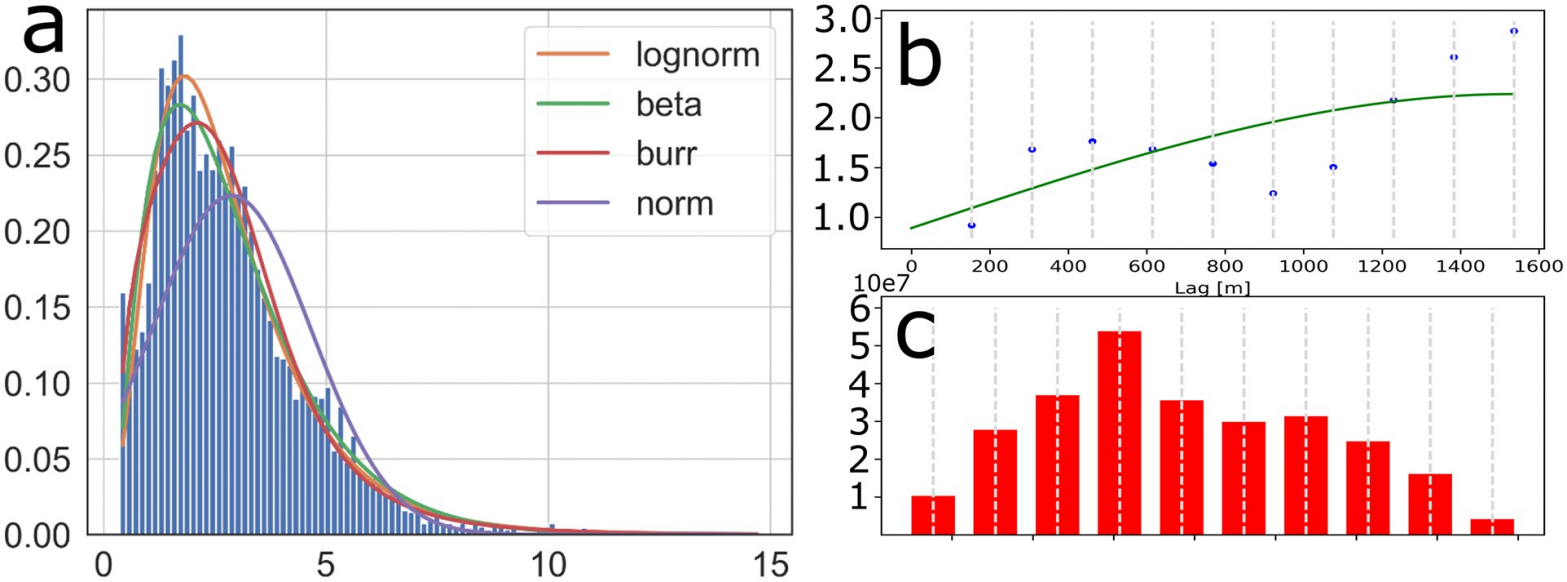
Statistical properties, (a) a histogram of raw CF data along with fitted log-normal, Burr, beta, and normal probability density functions for the entirety of the data set from the 28th of May 2022. Semivariogram (b), with data points plotted in blue dots and the a theoretical best fit function as a green line, and histogram (c) over number of samples for each lag bin, with each bar corresponding to a data point in (b). Semivariogram and histogram were generated using a random sample of 25% of the data.

Due to the “hot-spot” characteristics [2, 4, 17, 49] CF measurements in bloom conditions, we assumed that they would have a log-normal distribution. This assumption was confirmed by a best-fit analysis [50], evaluating log-normal, gamma, beta, Burr [51], and normal distributions (Table 3 and Fig 8a), using the sum of squared errors and the Akaike information criterion [50, 52]. From these analyses, the log-normal distribution had the lowest sum of squared errors, making it the best fit for the data.

**Table 3:** Best-fit analysis scores for the data set captured during the field trials using log-normal, gamma, beta, Burr and normal distributions as candidates.

Distribution	sum of squared errors	Akaike information criterion
log-normal	0.028270	878.292122
gamma	0.031805	926.462837
beta	0.032182	925.454839
Burr	0.037106	867.410520
normal	0.157564	1536.448687

In order to determine if the de-correlation lengths and variances used in the *ℓ*GP were reasonable, a semivariogram analysis of the data was performed (Fig 8b and 8c). In the semivariogram, a best fit spherical theoretical function [53] was plotted. From the semivariogram, two sills are visible, one at 500m, and one at 1600m, whereas the nugget semivariance appears to be 0.8, whereas the spatio-temporal variance lies between 2 and 3. From the best fit theoretical function, the parameters were found to be *M*_*x*_ = *M*_*y*_ = 1537m, $\sigma_{n}^{2}=0.90$, $\sigma_{c}^{2}=1.37$. Future *ℓ*GP variables can be picked from the semivariorgram, either from the fit theoretical function or the data, or an educated combination of the two.

## Discussion

We have shown that a 3D adaptive sampling network can provide near real time data updates to the operators, both in simulation and in field deployments. For the duration of the mission, the underwater vehicles in the network were able to adapt to and monitor the CF field within the operational volume. Acting on the data sent from the AUVs, the operators were able to measure and verify the predicted CF profile at the sampling location KB3 [42] and lab analysis, in Kongsfjorden, Svalbard.

The simulation study showed increased model accuracy and decreased model convergence time when using more than one autonomous vehicle within the same operational area. However, this was done using a static underlying field which could skew the results towards higher accuracy and precision than a dynamic underlying field. Despite of this, the findings hold true, the model accuracy increases with the number of vehicles in the same area, with a smaller increase in model accuracy per vehicle for each vehicle added. Further, the simulated CF field contained two large scale patches (Fig 4b), these did not mimic the small scale heterogeneity of the CF measurements observed in the field deployments, yet, the large scale patchiness carry similar characteristics. Using a sufficient number of vehicles, the RMSE converged towards the inherent process and sensor noise, and a lower value would be hard to achieve without changing the model parameters. The convergence of the model uncertainty and reduced prediction variability (Fig 6a and 6b) after 3000s in the field deployment indicate that the model was able to fit the field data. By using a Gaussian method, the model naturally became smoothed and “compressed”, not exhibiting the extremes of the measurements in the predictive mean. This effect is also visible in the field deployment data (Fig 5c and 5d). Further, by using an adaptive mission planning method, rather than a pre-planned one, the AUVs were able to seek the regions of highest variance and predicted CF values from the on board model. In having log-normally distributed data as input, the *ℓ*GPs posterior covariance, $\Sigma_{y}^{2}$, is directly dependent on the predictive mean, $\hat{y}$, and thus more exposed to variability in the field value, compared to the Gaussian random field [2, 54]. Adaptive methods have also been shown to be superior to the “lawnmower” maneuver [34] when measuring chlorophyll a.

Comparing the vertical profiles from AUVs, work boat, and *in vitro* measurements (Fig 7a), the measurements and predictions follow each other well below 20m. In the upper 15m, the work boat CF measurements fall out of the error bars of the prediction. For the discrepancy between model prediction and work boat CF measurements we suggest these possible origins; (1) inaccurate sensor calibration, (2) the model prediction uncertainty, erring on the positive side (Eq (6)), and (3) measurements were not co-located and contemporaneous. In comparing the predicted profiles (Fig 6c), there is little temporal development, and change in the prediction, indicating that even a small amount of data was sufficient to generate a representative prediction. The small surface discrepancy (Fig 7a), ≃ 0.1*μg*/*L*, between CF measruements and *in vitro* chlorophyll a can be explained by NPQ [45], a phenomenon in which the amount of measured CF and amount of intracellular chlorophyll a does not match the calibration of the sensor. Since the discrepancy is largest in the surface, where the light is strongest, NPQ is a likely candidate for explanation. There is also a temporal and vertical distance between work boat CF measurements and water sampling, increasing the probability of the discrepancy to originate from spatio-temporal process noise.

From the observations gathered during the field campaign, it was indicated that the predominant species of the algal bloom at the time of sampling was *Phaeocystis pouchetii*. Patches or colonies of *P. pouchetii* would be within one sampling envelope of a chlorophyll a fluorometer (≃ 3ml [55]), but would be integrated in the 10L Niskin bottle sample. This helps to explain the relative difference in noise level of the *in situ* CF measurements and the *in vitro* samples of chlorophyll a (Fig 7a). The values from *in vitro* samples also fits the predicted values from the *in situ* CF gathered by the AUVs, where the uncertainty reflects the measurement and process noise in the data. In addition, the *in vitro* samples were pseudoreplicated, with a variance of ≃ 0.01 [*μg*^2^/*L*^2^], within each sample. This is negligible in relation to the variance of the *in situ* CF measurements. The noise could also be caused by the low signal-to-noise ratio from fluorescence sensors, irradiance from the sun, different species of algae, photosynthetic rate, and health of the cells [56].

The assumption of a log-normal CF distribution [2, 17] was verified (Fig 8), however, the input to the *ℓ*GP is the cell-averaged CF values, not the raw measurements. When attempting a best-fit analysis on the segmented data, the results were less clear, with no obvious best fit distribution. A criticism to the best-fit analysis, is that the data is collected adaptively, seeking to maximize the CF value, and reduction in uncertainty (dependent on the CF value), thereby skewing the data in favour of higher values, thus skewing the histogram.

From the fit theoretical function in the variogram (Fig 8b), the nugget and variance was found to be within a reasonable range from the initial heuristic values. The nugget effect used was smaller than the one presented in the data, while the variance used was higher than the one used in the model. However, both values were within a reasonable range of the initial estimates. The horizontal de-correlation length predicted from the variogram, 1537m, is substantially longer than the de-correlation length used in the on board model, 600m. However, from the variogram data, there seems to be two predominant de-correlation lengths, the longer at 1537m and a shorter at around 500m. In choosing a shorter horizontal length scale, the model might over-estimate the uncertainty, but is more apt at preserving heterogeneity in the data. In the semivariogram, time was not taken into account, as the total mission time was less than two hours, and thus we can assume it to be fairly static [3, 4, 16].

## Conclusion

Our data shows that real-time data reported by multiple collaborating autonomous underwater vehicles combined with targeted water sampling can help increase our understanding of plankton patchiness. A method for adaptive tracking of CF based on a log-Gaussian process as a lightweight ocean model for a sensing network of AUVs and a boat was presented. The method models the chlorophyll a abundance, inferred by *in-situ* fluorescence measurements from made by the underwater vehicles. The AUVs were able to detect, explore, and measure the CF field inside the *operational volume* for the duration of the mission. The information relayed to the boat was acted upon and water samples and *in-situ* CF measurements were taken from the boat. By enabling on-board adaption, coupled with a small-bandwidth satellite communication, the vehicles were able to make decisions *in-situ*, targeting the desired combination of uncertainty reduction and CF maxima-seeking. By adding temporally increasing noise to the measurements, the on-board *ℓ*GP model was able to increase the significance of the most recent measurements. For the multi vehicle adaptive sampling with data sharing, the vehicles are forced to communicate, with the added benefit of allowing the operator to observe the data and communications in real time. Having an active operator, acting upon the information received, it was showed that targeted sampling of the water-column were enriched by the field estimates. In future work, including covariates, such as CTD, currents, spectral radiance, irradiance, reflectance from *in-situ* and remote sensing should be considered, providing a more holistic picture of the region of interest. Longer duration deployments in varying bloom conditions should be undertaken to further robustify the method by collecting data on spatial and temporal length-scales in a diverse set of scenarios, leading to a more well-calibrated model and utility function. Further work should also include a more formal configuration of the path-finding parameters in (Eq (8)), optimizing for the desired behavior in a multitude of bloom scenarios.

More information on pelagic biodiversity is urgently needed as the demand on these resources increases from climate change and harvesting. Future integration with automated eDNA samplers, such as LRAUV-ESP [57], or imaging instruments [58], together with adaptive sampling technologies reporting in real-time can be used to contextualize and inform plankton community sampling. This will help to fill important knowledge gaps and observe complex biological processes at fine-scale temporal and spatial resolutions.

## Supporting information

S1 File (CSV)

S2 File (CSV)

S3 File (CSV)

S4 File (CSV)

S5 File (CSV)
